# Enhancement of the Wear Particle Monitoring Capability of Oil Debris Sensors Using a Maximal Overlap Discrete Wavelet Transform with Optimal Decomposition Depth

**DOI:** 10.3390/s140406207

**Published:** 2014-03-28

**Authors:** Chuan Li, Juan Peng, Ming Liang

**Affiliations:** 1 Chongqing Key Laboratory of Manufacturing Equipment Mechanism Design and Control, Chongqing Technology and Business University, Chongqing 400067, China; E-Mails: chuanli@21cn.com (C.L.); juanpengcq@126.com (J.P.); 2 Department of Mechanical Engineering, University of Ottawa, ON K1N 6N5, Canada

**Keywords:** wear particle, oil debris sensor, monitoring, wavelet transform, optimal decomposition depth

## Abstract

Oil debris sensors are effective tools to monitor wear particles in lubricants. For *in situ* applications, surrounding noise and vibration interferences often distort the oil debris signature of the sensor. Hence extracting oil debris signatures from sensor signals is a challenging task for wear particle monitoring. In this paper we employ the maximal overlap discrete wavelet transform (MODWT) with optimal decomposition depth to enhance the wear particle monitoring capability. The sensor signal is decomposed by the MODWT into different depths for detecting the wear particle existence. To extract the authentic particle signature with minimal distortion, the root mean square deviation of kurtosis value of the segmented signal residue is adopted as a criterion to obtain the optimal decomposition depth for the MODWT. The proposed approach is evaluated using both simulated and experimental wear particles. The results show that the present method can improve the oil debris monitoring capability without structural upgrade requirements.

## 1. Introduction

The presence of wear particles in lubricating oil systems is an important index reflecting machine health conditions [1]. Oil debris sensors are thus effective tools for wear monitoring [2]. Because of its timely feedback, simple process and other economic advantages, the in-line oil debris sensor has been used for aircraft engine wear assessment and other applications [3]. Recently, different oil debris sensors such as the FerroScan^®^ sensor (GasTOPS Ltd. Ottawa, ON, Canada) and MetalScan^®^ monitor (GasTOPS Ltd.) have been developed and widely used for measuring the accumulation of the wear particles in lubricants [4].

**Figure 1.**
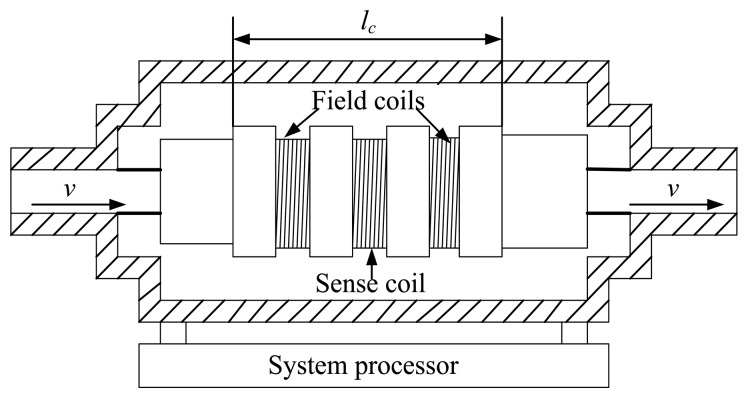
Schematic illustration of an oil debris sensor.

Usually an oil debris sensor is installed on a lubricating oil line. As shown in Figure 1, there are one sense coil and two field coils for an oil debris sensor. The two field coils are winded in the opposite directions, producing two opposing magnetic fields on the sense coil. By balancing the two magnetic fields, the strength of the excited magnetic field in the sense coil is close to zero. When a metallic (either ferromagnetic or non-ferromagnetic) particle flows through the sensor tube, the sense coil will produce an inductive electromotive force. Let *l_c_* denote the distance between the two ends of the field coils, and *v* the flow velocity of the oil. A signature similar to a 2π sinusoidal waveform that is a function of *l_c_* and *v* can be observed in the output signal of the sense coil in response to the pass of a metallic particle. For a constant velocity *v*, the amplitude of the particle signature is proportional to the mass of the ferromagnetic particle or the surface area of the non-ferromagnetic particle [5,6]. Therefore, the wear condition can be estimated by examining the particle signature [7]. As such, on-line wear measurement of the oil-lubricated mechanical systems can be done by monitoring the amount and the size of the metallic particles on a real time basis, providing that high-quality particle signals are available.

In reality, the wear debris signal obtained from a sensor is often polluted by background noises from electric sources such as a data acquisition system and by the vibration interferences from mechanical sources, e.g., the moving parts of the machine being monitored and the neighboring machines. The background electric noise is unavoidable for all electric sensors. As for the vibration interferences, a brief analysis can explain its generation mechanism. When the ambient vibration drives the sense coil to cut through the magnetic field of the eddy currents, an electromotive force *E*(*t*) following Faraday's law is generated as:

(1) $$E(t)=Bl_{c}\frac{\text{d}x(t)}{\text{d}t}$$

where *B* denotes the magnetic field, *x*(*t*) is the vibration displacement of the sense coil. According to Equation (1), the output voltage of the vibration interference is approximately proportional to the vibration velocity. Hence the output signal of the oil debris sensor manifests the combined effect of particle signature, electric noise and vibration velocity characteristics. The target particle signal can be therefore distorted by both the noises and interferences [8]. The situation is even worse for fine particles whose weak signatures are not only subjected to shape distortion but also being completely immersed deep in the noise. As the small wear particles are very important for the early wear assessment, extracting weak particle signatures from the contaminated sensor outputs becomes a challenging task for the wear assessment of mission-critical systems [9].

Both hardware- and software-based approaches have been developed to enhance the wear particle monitoring performance for the oil debris sensors. Within the hardware framework, the three-coil structure has achieved good anti-noise effects [5]. However, the fixed hardware structure is ineffective in response to variable environmental noises and interferences. Several software-based methods have been reported for extracting the metallic particle signatures from oil debris sensor signals. Among them the threshold method is a very popular approach. With this approach, a metallic particle signature is confirmed if the amplitude of the sensor output signal is above a preset threshold value. This is obviously valid only if the signal-to-noise ratio (SNR) of the signal is sufficiently high. As an effective tool for signal denoising [10], wavelet transform has been widely used for different cases [11–13].

On the basis of the traditional threshold method, Yi *et al.* [14] suggested an improved wavelet thresholding technique signal noise smoothing. Galiana *et al.* [15] proposed a wavelet packet transform de-noising method with adaptive thresholds. Hong and Liang [16] improved the performance of the oil debris sensor using a fractional calculus technique. Fan *et al.* [17] proposed a joint time-invariant wavelet transform and kurtosis (TIWT) approach to wear particle detection in a noisy environment. Bozchalooi and Liang [18] developed an adaptive subband filtering approach for in-line identification of wear particle signals. The above wavelet-based methods have made important contributions to metallic particle signature extraction from oil-lubricated systems. However, optimal decomposition depth of the wavelet transform in these studies has not been adequately addressed. Though Fan *et al.* [17] specified an upper bound of decomposition levels, the best number of decomposition levels remains to be determined. The optimization of decomposition depth is very important in the context of the sensor signal processing. This is because over-decomposition tends to distort the particle signature measurement whereas on the other hand insufficient decomposition may leave fine particles undetected. To measure fine particle with minimal distortion, Li and Liang [5] reported an integral enhanced empirical mode decomposition (EMD) and correlated reconstruction approach, which has a good potential for better estimating metallic particle sizes for the wear particle monitoring. Due to its intrinsic excess computing requirement, nevertheless, the EMD-based approach is not convenient for real-time implementations [19,20]. In comparison to the EMD-based approach, the wavelet method is better suited to on line deployment, if the optimal decomposition depth can be specified to ensure the minimum signature distortion [21]. As the optimal decomposition depth is different for different sensor outputs and wear conditions, it is desirable to develop a methodology to quickly determine the optimal decomposition depth for a given oil debris sensor signal.

For this purpose, we suggest using the maximal overlap discrete wavelet transform (MODWT) with the optimal decomposition depth to extract authentic metallic particle signatures from the oil debris sensor signals. The MODWT features invariant wavelet coefficient and scale coefficient of translation [22–24]. This means that all decomposition levels deliver the same time resolution. Therefore the MODWT is appropriate for detecting the existence of the particle in different decomposed levels. To measure wear particle signatures with minimal distortion, the root mean square deviation of kurtosis of the segmented signal residue (RMSDK_SSR) is proposed as a criterion to find the optimal decomposition depth of the MODWT. In this way we can locate the optimal decomposition depth adaptively to measure particle signatures as reliably as possible, and therefore enhancing the wear particle monitoring capability within a software framework and without structural upgrade requirements.

## 2. The Proposed MODWT-ODD Approach

### 2.1. Overview of the MODWT

The MODWT can be regarded as a modified version of discrete wavelet transform (DWT). Consider a signal **x** = {*x_t_*; *t* = 0, …, *n* − 1} where *n* is a power of 2. The DWT of **x** is illustrated as follows [25]. Here we denote a low-pass scaling filter by f = {*f_j_*; *j* = 0, *…*, *l* − 1} and a high-pass wavelet filter as k = {*k_j_*; *j* = 0, …, *l* − 1}, where *l* is the length of the filter. For a nonzero integer *m*, the low-pass filter can be defined based on:

(2) $$\sum_{j=0}^{l-1}{f_{j}}^{2}=1,\text{and}\sum_{j=0}^{l-1}f_{i}f_{j+2m}=\sum_{j=-\infty }^{\infty }f_{i}f_{j+2m}=0$$

The high-pass filter can also be defined similarly. In addition, both the high-pass and the low-pass filters must be quadrature mirror filters satisfying:

(3) $$k_{j}={\left(-1\right)}^{j}f_{l-j-1}$$

(4) $$f_{j}={\left(-1\right)}^{j+1}k_{l-j-1}$$

For the *i*th scale, let transform coefficients **W** = {*W_i_*_−1_,*_t_*; *t* = 0, …, *n_i_*_−1_ − 1}, with *n_i_ = n/*2*^i^*, and *W*_0_,*_t_* = *x_t_*,. According to the pyramid algorithm [26], the wavelet and the scaling coefficients of the DWT at the *i*th scale are respectively given by:

(5) $$w_{i,t}=\sum_{j=0}^{l-1}f_{j}w_{i-1,(2t+1-j)\mathrm{mod}(n_{i}-1)},(t=0,\ldots ,n_{i}-1)$$

(6) $$z_{i,t}=\sum_{j=0}^{l-1}k_{j}w_{i-1,(2t+1-j)\mathrm{mod}(n_{i}-1)},(t=0,\ldots ,n_{i}-1)$$

where “mod” denotes the modulus after division.

As illustrated in the above equations, the sample size of signal **x** for the *i*-level DWT decomposition must be an integer multiple of 2*^i^*. On the contrary, there is no such a sampling limit for the MODWT. The relationships of the scale filter (*f̃_j_*) and the wavelet filter (*k̃_j_*) of the MODWT with their DWT counterparts are:

(7) $${\tilde{f}}_{j}=f_{j}/\sqrt{2}$$

(8) $${\tilde{k}}_{j}=k_{j}/\sqrt{2}$$

Substituting Equation (2) into Equations (7) and (8) leads to:

(9) $$\sum_{j=0}^{l-1}{{\tilde{f}}_{j}}^{2}=1/2,\text{and}\sum_{j=0}^{l-1}{\tilde{f}}_{j}{\tilde{f}}_{j+2m}=\sum_{j=-\infty }^{\infty }{\tilde{f}}_{j}{\tilde{f}}_{j+2m}=0$$

Likewise, combining Equations (3), (4) and (9) yields:

(10) $${\tilde{k}}_{j}={\left(-1\right)}^{j}{\tilde{f}}_{l-j-1}$$

(11) $${\tilde{f}}_{j}={\left(-1\right)}^{j+1}{\tilde{k}}_{l-j-1}$$

To avoid the downsampling issue of the DWT, the MODWT delivers a new filter at each level, by inserting *2^i^*^−1^ − 1 zeros into each level of { (*f̃_j_*)} and {(*k̃_j_*)} [27]:

(12) $$\left\{{\tilde{f}}_{j}\}=\{{\tilde{f}}_{0},0,\ldots ,0,{\tilde{f}}_{1},0,\ldots ,0,\ldots ,{\tilde{f}}_{l-2},0,\ldots ,0,{\tilde{f}}_{l-1}\right\}$$

(13) $$\left\{{\tilde{k}}_{j}\}=\{{\tilde{k}}_{0},0,\ldots ,0,{\tilde{k}}_{1},0,\ldots ,0,\ldots ,{\tilde{k}}_{l-2},0,\ldots ,0,{\tilde{k}}_{l-1}\right\}$$

Similar to the pyramid algorithm of the DWT, the wavelet and the scaling coefficients of the MODWT at the *i*th scale can be expressed as follows:

(14) $$w_{i,t}=\sum_{j=0}^{l-1}{\tilde{f}}_{j}w_{i-1,(t-2^{i-1})\mathrm{mod}(n)},(t=0,\ldots ,n-1)$$

(15) $$z_{i,t}=\sum_{j=0}^{l-1}{\tilde{k}}_{j}w_{i-1,(t-2^{i-1})\mathrm{mod}(n)},(t=0,\ldots ,n-1)$$

The Fejer-Korovkin (FK) mother wavelet proposed by Nielsen [28] is capable of optimal frequency localization and has less energy leakage for signal decomposition. Moreover, the shape of the FK wavelet is similar to that of the Daubechies wavelet which has been widely used in extracting sinusoidal signals. For the above reason, the 22-order FK wavelet is selected as the mother wavelet of the MODWT.

It is worth noting that the length of the coefficients at each level is 2*n*. This means that the data length *n* is doubled after the decomposition by the MODWT. The data length will be restored after the signal reconstruction. This process will be described in the forthcoming subsection.

Due to the translation invariant wavelet coefficients and scale invariance, all decomposition levels of the MODWT feature the same time resolution without phase distortion. Therefore, in this research the MODWT is employed to detect the metallic particle signatures. One of the key parameters for the MODWT is the number of the decomposition levels (*i.e.*, decomposition depth). In the following subsection we will examine the influence of the decomposition depth on the capability of the wear particle signature monitoring in detail.

### 2.2. Influence of the Decomposition Depth on Particle Signature Integrity

Though the increase in the decomposition depth is desirable to identify particle signatures from noisy signals, it leads to the reduced band width of each scale as well as excessive computation burden. For this reason, Fan *et al.* [17] suggested the following equation to calculate the maximum level for wavelet transform:

(16) $$L=\frac{\ln s_{f}-\ln f_{\min}}{\ln2}-1$$

where *L* is the maximum decomposition depth, *s_f_* the sampling frequency, *f*_min_ the lowest frequency of particle signature corresponding to the lowest oil velocity *v*_min_, *i.e.*, *f*_min_ *= v*_min_*/l_c_* with *l_c_* being the distance between the two ends of the field coils, as marked in Figure 1.

**Figure 2.**
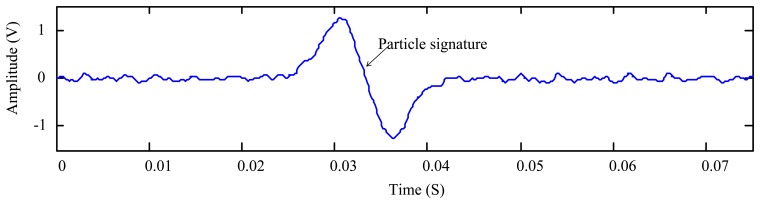
Signal obtained from an oil debris sensor with a single particle (enlarged view in temporal scale).

The above equation presents an upper limit of the decomposition depth. However, it is not the optimal in general. For a sensor signal with a high level SNR, the particle can be measured with a few decomposition levels that could be much less than the *L* given by Equation (16). In this case, the signature coefficients spread to higher levels may be smaller than the threshold and therefore are omitted by the thresholding. In other words, the excessive decomposition is obviously overkill and will cause the signature distortion for the wear particle monitoring. This can be illustrated using a simulation example. To simulate the output signal of the oil debris sensor, we first observe the real sensor output as plotted in Figure 2 in response to the appearance of a metallic particle.

As is introduced in Section 1 and shown in Figure 2, the metallic particle signature is similar to a 2π sinusoidal waveform that can be simulated by:

(17) $$s(t)=\{\begin{array}{cc} b\sin(2\pi f(t-T_{0})); & T_{0}\leq t\leq T_{0}+\frac{1}{f}\\ 0; & T_{a}\leq t\leq T_{0}\&T_{0}+\frac{1}{f}<t<T_{b} \end{array}$$

where *b* is the signature amplitude that is proportional to the particle size, [*T*_0_, *T*_0_ + 1/*f*] the duration of the particle signature, and [*T_a_*, *T_b_*] the observation time interval. So one can measure the wear particle size by determining the particle signature amplitude of the sensor output signal.

**Figure 3.**
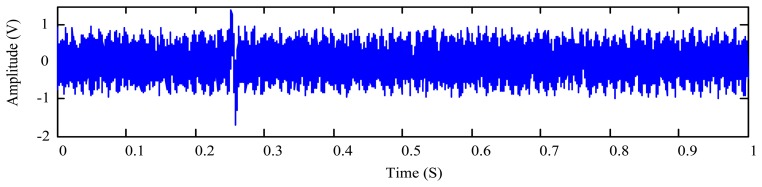
Simulated oil debris sensor signal defined by Equation (18).

Besides the particle signature, the signal from the sensor is usually contaminated by the vibration interference and random background noise. Considering the above factors, a simulated sensor output signal *x*(*t*) is expressed as:

(18) $$x(t)=s(t)+0.2\sin(987\pi t)+0.3\sin(817\pi t)+x_{0}(t)$$

where [*T_a_*, *T_b_*] = [0, 1], *b* = 0.8, *f* = 80 Hz, *T*_0_ *=* 0.25, *x*_0_(*t*) is a random number in the range [−0.5, 0.5], and sampling frequency is set as 8,000 Hz. The four items of the right-hand side of Equation (18) represent the particle signature, the two vibration interference components, and the background noise, respectively. The temporal waveform of the simulated signal is plotted in Figure 3.

**Figure 4.**
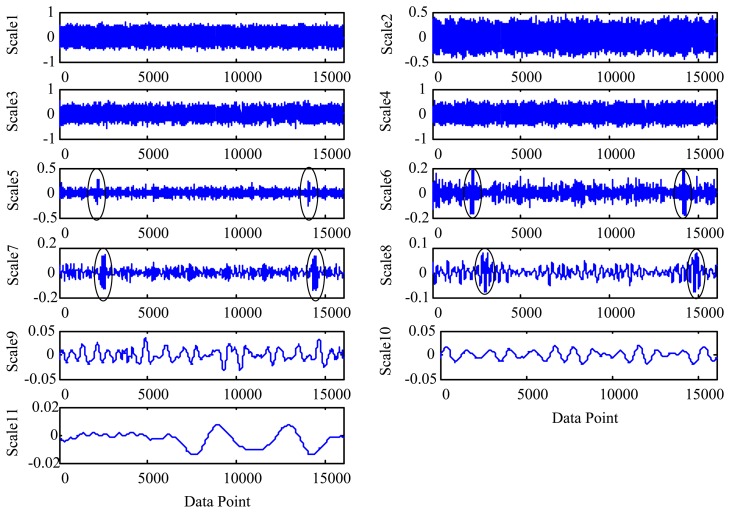
The MODWT coefficients of each scale. For convenience, the profile coefficients of the scale 10 are marked as “scale 11”. At each scale, coefficients related to particle signatures are highlighted using circles.

To illustrate, we consider an oil debris sensor with a bore diameter of 3/8 inch. Assuming the minimum oil velocity is 0.12 m/s and the distance between the two ends of the field coils is 0.03 m, then based on Equation (16) the maximum decomposition depth *L* is 10. The MODWT is employed to decompose the simulated signal to the depth of 10 levels. Figure 4 shows the decomposed wavelet detail coefficients for scales 1 to 10. For convenience, the profile coefficients of the scale 10 are marked as “scale 11”. It is shown that, due to the padding of zeros as illustrated in Equations (12) and (13), the wavelet coefficient length for each scale is double of that of the original signal *x*(*t*). As introduced in Section 2.1, the data length *n* is doubled as 2*n* after the decomposition by the MODWT. So one signature (sinusoidal) waveform as shown in Figure 3 is symmetrically doubled as two signature waveforms as shown in Figure 4. From Figure 4 one cannot identify the particle signature coefficients until the 5th scale. However, if the decomposition level is greater than 7, the particle coefficients yield confusing results (reflected as the time-shift of the particle footprint) owing to the over-decomposition.

**Figure 5.**
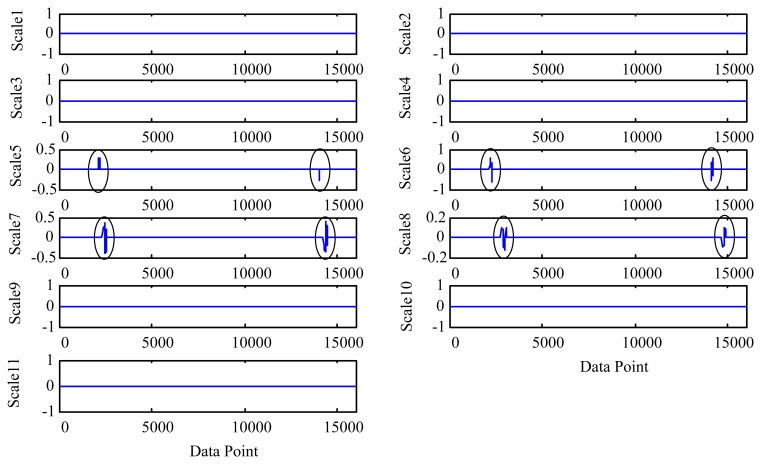
Wavelet coefficient shrinkages for all scales. For convenience, the profile coefficients of the scale 10 are marked as “scale 11”. At each scale, coefficients related to particle signatures are highlighted using circles.

It should be noted that the over-decomposition itself does not cause the error in our case. Instead, the signature measurement distortion mainly owes to the thresholding steps as introduced as follows. The above MODWT results will be denoised for the particle signature measurement (this is the representation of the wear measurement in the electric domain). This can be done by hard thresholding using the universal threshold $\lambda =\sigma \sqrt{2\log(N)}$ where *σ* is the noise level calculated separately for each scale and *N* represents the signal length [29]. Figure 5 shows the denoised (*i.e.*, shrunk) wavelet coefficients at each scale. Note that the wavelet coefficients at levels 1 to 4 and 9 to 11 are removed completely because of the lack of relevance to the target signature. It should be particularly noted that the scales higher than level 8 have also the signature coefficients which are completely removed by the thresholding steps, as they are smaller than the universal threshold in higher scales. This suggests that excess decomposition depth does not contribute to further improvement of the signature quality, but sacrifices parts of coefficients which are spread to higher scales.

**Figure 6.**
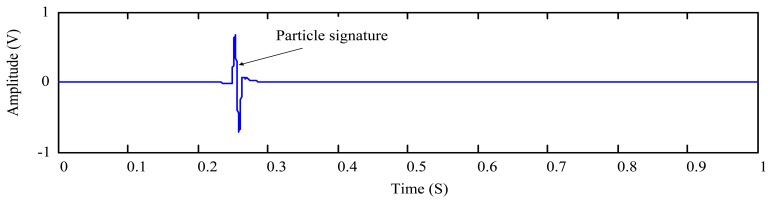
The extracted particle signature reconstructed from the shrunk coefficients as shown in Figure 5.

The shrunk coefficients are subsequently used to reconstruct the signal which more clearly displays the particle signature *s*_1_(*t*) (Figure 6).

Figure 6 shows that the amplitude of the extracted particle signature *s*_1_(*t*) is around 0.7 (+0.6622, −0.7076). The true amplitude, in contrast, is 0.8 as given by Equation (18). This indicates that an error could be introduced due to the overkill of the signature coefficients scattered at the higher scales, resulting from the excess decomposition depth used in the MODWT. To quantitatively evaluate the measurement error, Euclidean distance is employed here to illustrate the similarity of the two signals *s*(*t*) and *s*_1_(*t*) [30–32]. It has been proven that the smaller the Euclidian distance is, the more similar the two signals will be [33]. The Euclidean distance between the simulated particle signature *s*(*t*) and the extracted signature *s*_1_(*t*) is given by [34]:

(19) $$d(s(t),s_{1}(t))=\sqrt{\sum_{t=T_{a}}^{T_{b}}{\left(s(t),s_{1}(t)\right)}^{2}}$$

Using the above equation, the Euclidean distance between the two signals is 1.3151. Obviously, if the two signals are identical, the Euclidean distance will be zero. This suggests that excessive decomposition depth leads to distorted signature and greater measurement error. On the other hand, if the decomposition depth is too shallow (e.g., less than 3 for the given example as shown in Figures 4 and 5), the MODWT will not be able to detect the existence of the metallic particle. This implies the existence of an optimal decomposition depth at which the particle signature can be detected with the minimum distortion as validated in Section 3. Therefore an appropriate decomposition depth is very important for reliable measurement of the wear particle and its sensor output signature.

Theoretically, the Euclidean distance given by Equation (19) could be used as a criterion to find the optimal decomposition depth *L*_opt_ of the MODWT. A decomposition depth related to the minimal Euclidean distance would be considered as the optimal depth *L*_opt_. Using this optimal depth, one can extract the best particle signatures for a given sensor output signal. Unfortunately, the application of the Euclidean distance requires prior knowledge of the ‘true’ particle signature (*i.e.*, *s*(*t*) shown in Equation (19)). For the simulation case as illustrated above, the ‘true’ particle signature *s*(*t*) can be considered available but it is not the case for real applications because ‘true’ signature is exactly what we are looking for. If we knew the “true” particle signature, we would not need to do anything further. Hence the Euclidean distance criterion cannot be used to find the optimal decomposition depth in reality.

After extracting the particle signatures, the residue signal can reflect the extraction effect (the measurement precision) to a certain extent. Generally speaking, if the residue is sufficiently short, it can be regarded as in a steady state [35]. If the particle signature is over or under extracted, the steady state of the residue signal will be disturbed. In other words, the extraction should be good enough if the steady state of the residue is the same as the particle-free sensor signal. Considering the short-term stationarity of the residue signal, we adopt the root mean square deviation of kurtosis value of the segmented signal residue (RMSDK_SSR), as the criterion to evaluate the steady state of the residue signal and to identify the optimal decomposition depth *L*_opt_. The details of this criterion are described in the following subsection.

### 2.3. The RMSDK_SSR Criterion

As illustrated above, the Euclidean distance can be used to measure the similarity between the extracted and the true particle signatures for simulated signals. However, due to the unavailability of the authentic particle signature, the Euclidean distance is not a relevant measure for real signals. It is desirable that the extracted particle signature contains as much the true signature content as possible and as little background noise as well interference contents as possible. However, it is difficult to measure the quality of the extracted signature directly. Hence, the residue signal, *i.e.*, the difference between the raw signal and the extracted particle signature, is used for this purpose. To this end, we propose the RMSDK_SSR criterion to replace the Euclidean distance measure for real signals. This criterion is explained as follows.

For a raw signal *x*(*t*) and the extracted particle signature *s*_1_(*t*), *t* ∈ [*T_a_*, *T_b_*], the residue signal *s*_0_(*t*) is given by:

(20) $$s_{0}(t)=x(t)-s_{1}(t)$$

Denoting the length of a signal segment by *T_s_*, the number of such segments, *k*, for the residue signal *s*_0_(*t*) is calculated by:

(21) $$k=\left\lfloor (T_{b}-T_{a})/T_{s}\right\rfloor$$

where “└. ┘” is the round-down operator. For the *i*-th full-length (*T_s_*) segment of the residue signal $s_{0}^{i}(t),i\in [1,m]$, its kurtosis is expressed as:

(22) $$\text{kur}(s_{0}^{i}(t))=\frac{\frac{1}{T_{s}}\sum_{j=T_{i}}^{T_{i}+T_{s}}{\left(s_{0}^{i}(t_{j})-{\bar{s}}_{o}^{i}\right)}^{4}}{\frac{1}{T_{s}}\sum_{j=T_{i}}^{T_{i}+T_{s}}{\left({\left(s_{0}^{i}(t_{j})-{\bar{s}}_{0}^{i}\right)}^{2}\right)}^{2}}$$

where *kur*(.) represents the kurtosis value, *T_i_* and *T_i_*+*T_s_* denote the lower and upper boundaries of the *i*-th segment of the residue, and ${\bar{s}}_{0}^{i}$ is the mean of the *i*-th segment of the residue.

To measure the deviation of the *k* kurtosis values $\text{kur}(s_{0}^{1}(t))$, $\text{kur}(s_{0}^{2}(t))$, …, $\text{kur}(s_{0}^{i}(t))$, …, $\text{kur}(s_{0}^{k}(t))$ from their mean value, we propose to use the root mean square deviation of kurtosis value of the segmented signal residue (RMSDK_SSR), *i.e.*,

(23) $$\text{RMSDK}\_\text{SSR}(x(t),s_{1}(t))=\sqrt{\frac{1}{k}\sum_{i=1}^{k}{\left(\text{kur}\left(s_{0}^{i}\left(t\right)\right)-\bar{\text{kur}}\right)}^{2}}$$

where $\bar{\text{kur}}$ is the mean of the *k* kurtosis values.

Having defined the RMSDK_SSR criterion, we illustrate hereafter that this criterion is capable of evaluating the quality of the extracted particle signature.

#### Lemma 1

For a wear particle signal *x*(*t*) and its two extracted metallic particle signatures $s_{1}^{\prime }(t)$ and $s_{1}^{\prime \prime }(t)$, if the background noise and the interferences are of short-term stationary, and $\text{RMSDK}\_\text{SSR}(x(t),s_{1}^{\prime }(t))<\text{RMSDK}\_\text{SSR}(x(t),s_{1}^{\prime \prime }(t))$, the quality of the level of integrity of the extracted signature $s_{1}^{\prime }(t)$ is better than that of $s_{1}^{\prime \prime }(t)$.

##### Proof

Since the background noise and the interferences are of short-term stationary, the kurtosis of the background noise and interferences should be unrelated to the data length. In other words, the kurtosis of the part of residue signal is equal to that of the whole residue signal. If the particle signature *s*_1_(*t*) is completely extracted without any distortion, one has:

(24) $$\text{kur}(s_{0}^{i}(t))=\text{kur}(s_{0}(t))$$

Substituting Equation (24) into Equation (23) leads to:

(25) $$\text{RMSDK}\_\text{SSR}(x(t),s_{1}(t))=0$$

On the other hand, certain peaks and/or valleys will appear in the residue signal if the particle signature is not properly extracted (either over or under extracted). The short-term stationarity of the residue signal is therefore disturbed by those peaks and valleys. For a residue segment $s_{0}^{i^{\prime }}(t)$ corresponding to those peaks and valleys, we have:

(26) $$\text{kur}(s_{0}^{i'}(t)\neq \text{kur}(s_{0}(t))$$

and accordingly:

(27) $$\text{RMSDK}\_\text{SSR}(x(t),s_{1}^{'}(t))>0$$

If $\text{RMSDK}\_\text{SSR}(x(t),s_{1}^{\prime }(t))<\text{RMSDK}\_\text{SSR}(s(t),s_{1}^{\prime \prime }(t))$, the derivation of the series ( $\text{kur}(s_{0}^{1^{\prime \prime }}(t))\text{kur}(s_{0}^{2^{\prime \prime }}(t))$, …, $\text{kur}(s_{0}^{i^{\prime \prime }}(t))$, …, $\text{kur}(s_{0}^{k^{\prime \prime }}(t))$) is greater than that of the series ( $\text{kur}(s_{0}^{1^{\prime }}(t))$, $\text{kur}(s_{0}^{2^{\prime }}(t))$, …, $\text{kur}(s_{0}^{i^{\prime }}(t))$, …, $\text{kur}(s_{0}^{k^{\prime }}(t))$). Considering the fact that kurtosis is measure of impulsiveness or peakedness of a signal, the aforementioned condition indicates that the peakedness in the residue signal $s(t)-s_{1}^{\prime \prime }(t)$ is greater than that in $s(t)-s_{1}^{\prime }(t)$. This proves that the integrity of $s_{1}^{\prime }(t)$ is better than that of $s_{1}^{\prime \prime }(t)$.

Consequently, the RMSDK_SSR is a suitable statistics criterion to quantify the integrity of the extracted particle signature. Thus, we can use the MODWT to decompose the sensor output signal from the second level up to the maximum level *L* (Equation (16). For each decomposition depth, a signature candidate is obtained after the thresholding and the reconstruction operations. Hence there are *L*-1 signature candidates available for evaluation. The RMSDK_SSR value is then calculated for each signature candidate using Equation (23). According to Lemma 1, the decomposition depth associated with the minimal RMSDK_SSR of the *L*-1 values is chosen as the optimal one, *i.e.*, *L*_opt_.

### 2.4. Specification of the Segment Length T_s_ for RMSDK_SSR Computation

To compute the RMSDK_SSR values, the proper segment length, *T_s_*, has to be specified to more accurately determining the optimal decomposition depth. Theoretically, a long segment would make the short-term stationarity assumption invalid. Though a smaller *T_s_* is preferred for better examining the peaks and valleys in the residue signal, too small a *T_s_* will cause the calculation error of kurtosis, as given by Equation (22). Hence, it is suggested that the segment length should cover the duration of a particle signature, *i.e.*,

(28) $$T_{s}\leq 1/f_{\max}$$

where *f*_max_ represent the highest frequency of particle signal corresponding to the largest oil velocity *v*_max_.

### 2.5. Procedure of the Proposed Approach

**Figure 7.**
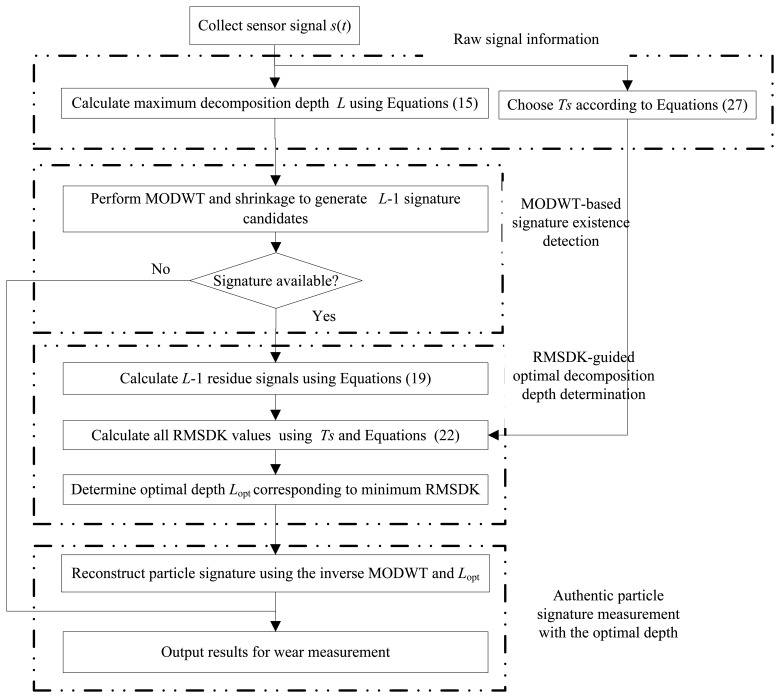
Flowchart of the proposed MODWT-ODD approach.

With the above discussions, the proposed MODWT-ODD approach can be summarized in a flowchart shown in Figure 7 and detailed as follows.

Step 1. Collect the measurement signal *s*(*t*) from the wear debris sensor.
Step 2. Calculate the maximum decomposition depth *L* and select the segment length *T_s_* according to Equations (16) and (28), respectively.
Step 3. Perform *L* − 1 times of MODWT shrinkages and reconstructions to generate *L* − 1 particle signature candidates. If no candidate available, go to Step 6.
Step 4. Calculate *L* − 1 residue signals and their corresponding RMSDK_SSR values using Equations (20) and (23), respectively.
Step 5. Identify the decomposition depth related to the minimum RMSDK_SSR value as the optimal decomposition depth *L*_opt_. The signature candidate corresponding to *L*_opt_ is the final extracted particle signature.
Step 6. Output the particle signature for the wear particle measurement. End.

## 3. Evaluation of the Proposed Approach Using Simulated Signal

For real applications, the real particle signature is unknown, and hence it is difficult to directly examine the effectiveness of the RMSDK_SSR criterion for real applications. In this section, a simulated particle signature is added into simulated noise and interference components. The proposed method is then employed to measure the particle signature. The effectiveness of the present approach can therefore be validated by comparing its result with the Euclidean distance between the measured and the original particle signatures. To this end, the simulated sensor signal *x*(*t*) given by Equation (18) is again used in this section.

As discussed in the previous section, the Euclidean distance has been demonstrated to be an effective criterion in determining *L*_opt_ for simulated sensor signals (though it cannot be used for real signals) and hence is used to find *L*_opt_ for *x*(*t*). Different decomposition depths lead to different extracted signatures via the MODWT. For a decomposition of depth *j*, the Euclidean distance *d*(*s*(*t*), *s_j_*(*t*)) between the added particle signature *s*(*t*) and the extracted signature *s_j_*(*t*) can be calculated using Equation (19). Table 1 lists the results for nine different decomposition depths (Note: the lowest depth of decomposition in Table 1 is two because the result of decomposition of depth 1 is the signal itself). As shown in this table, the shortest Euclidean distance is *d*(*s*(*t*), *s*_5_(*t*)). This indicates that *L*_opt_ = 5 for the simulated sensor signal.

The RMSDK_SSR criterion is then used to obtain the optimal decomposition depth *L*_opt_ for the MODWT of *x*(*t*). For each decomposition depth, one can also calculate *RMSDK_SSR*(*x*(*t*),*s_i_*(*t*)) using Equation (25) with *T_s_* = 0.01 s. For comparison, the RMSDK_SSR values for all decomposition depths are also displayed in Table 1 where the smallest RMSDK_SSR is *RMSDK_SSR*(*x*(*t*),*s*_5_(*t*)). Comparing to the Euclidean distance values, the RMSDK_SSR values are more close to each other. To better compare different decomposition depths and for fair comparison of the two criteria, a relative normalization method is proposed here. The relative normalization can be illustrated as follows. For a vector *a* = [*a*_2_, *a*_3_, …, *a_L_*] consisting of *L*-1 components, let *a*_max_ denote the maximum component and *a*_min_ the minimum one. Then relatively normalized value of element *a_p_* is:

(29) $$\bar{\bar{a_{p}}}=\frac{a_{p}-a_{\min}}{a_{\max}-a_{\min}},(p=2,3,\ldots ,L)$$

where the hat “=” denotes the relative normalization operator, a_max_ = max[a_n_ | *n* = 2,3, …,*L*], and a_min_ = min[a_n_ | *n* = 2,3, …,*L*].

**Figure 8.**
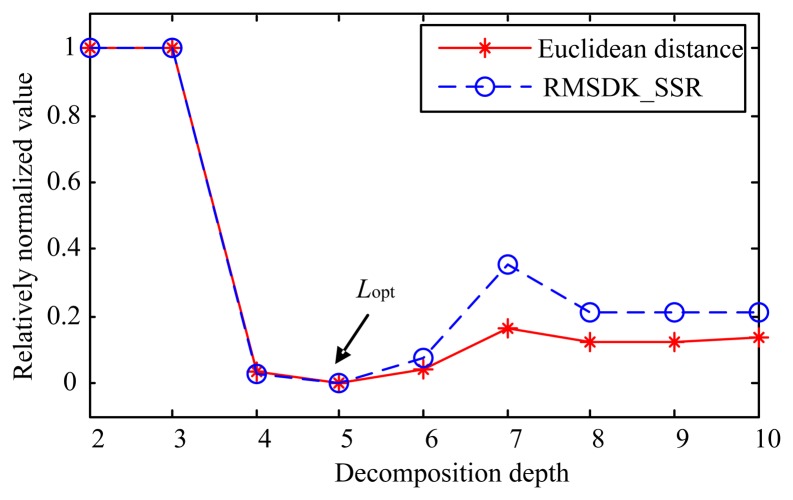
Relatively normalized Euclidian distance and RMSDK_SSR.

**Figure 9.**
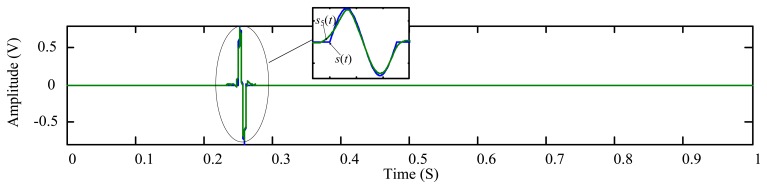
Comparison between the added and the extracted particle signatures.

By applying the relative normalization to *d*(*s*(*t*),*s_i_*(*t*)) and *RMSDK_SSR*(*x*(*t*),*s_i_*(*t*)) values in Table 1, one can plot $\bar{\bar{d(s(t),s_{j}(t))}}$ and $\bar{\bar{\text{RMSDK}\_\text{SSR}(x(t),s_{j}(t))}}$ as shown in Figure 8. One may notice that *s*(*t*) and *s_j_*(*t*) are used for calculating the Euclidean distance, while *x*(*t*) and *s_j_*(*t*) for RMSDK_SSR. This again proves that the Euclidean distance criterion cannot be used in reality. According to Figure 8, we can see that the relatively normalized Euclidean distance and RMSDK_SSR display similar patterns but the RMSKD_SSR criterion shows a higher sensitivity when the depth of decomposition increases. Both criteria indicate that the optimal depth is 5, *i.e.*, *L*_opt_ = 5. The worst decomposition depths are 2 and 3 levels where no candidate signature is detected. The extracted particle signature *s*_5_(*t*) is plotted in Figure 9 in which the original simulated signature *s*(*t*) is also plotted for comparison.

According to Table 1, one can see that both the Euclidean distance criterion and the RMSDK_SSR criterion are effective to determine the optimal decomposition depth *L*_opt_ for simulated sensor output signals consisting known particle signatures. However, the Euclidean distance criterion identifies the optimal depth by comparison with the “true” particle signature whereas the RMSDK_SSR criterion does so without knowing the details of the “true” signature. Therefore the Euclidean distance criterion cannot be used for real applications. On the contrary, as given by Equation (19), the residue signal *s*_0_(*t*) is all that is needed to calculate the RMSDK_SSR value and to identify the optimal decomposition depth. Through evaluating the short-term stationarity of the residue signal *s*_0_(*t*), the RMSDK_SSR is capable of measuring the integrity of the extracted wear particle signature *s*_1_(*t*). The higher the level of integrity of the measured signature *s*_1_(*t*) is, the smaller the RMSDK_SSR value will be. Therefore, the proposed RMSKD_SSR criterion for identifying the optimal decomposition depth is validated both theoretically and numerically via simulation.

## 4. Experimental Evaluations

**Figure 10.**
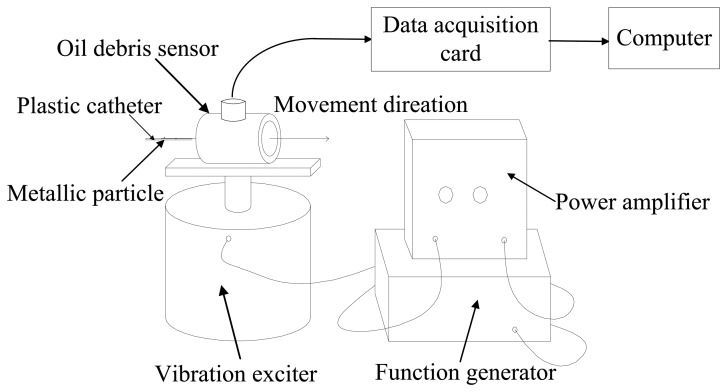
Experimental set-up.

In this section, the proposed MODWT-ODD approach is used to monitor real wear metallic particles from experimental signals. As shown in Figure 10, a vibration exciter (4809, Bruel & Kjaer, Nærum, Denmark) was used to produce the vibration interferences controlled by a function generator and a power amplifier (2706, Bruel & Kjaer). A 3/4″ oil debris sensor was mounted on top of the vibration exciter. The output signal of the sensor was fed into a computer for particle signature extraction via a data acquisition card (AT-MIO-16DE-10, NI, Austin, TX, USA).

Two aluminum wear particles (508 μm and 125 μm in diameter respectively) and one titanium wear particle of 70 μm in diameter were collected and implanted into three plastic catheters respectively. For each experiment, each of the catheters was manually moved through the tube of the oil debris sensor to generate signal which is a mixture of adjustable vibration interferences, random noises and particle signatures. The sampling frequency is 8,000 Hz.

### 4.1. Metallic Particle Signature Monitoring under Lower SNR Condition

**Figure 11.**
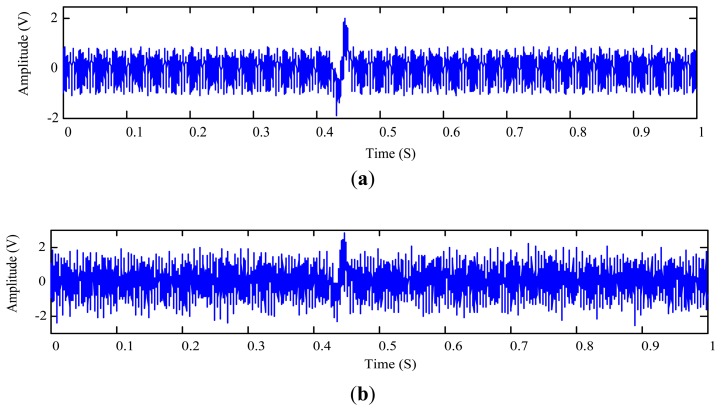
Particle signature of the 508 μm aluminum particle: (**a**) sensor output signal *r*_1_(*t*); and (**b**) mixed signal *x*(*t*) consisting of *r*_1_(*t*) and *r*_2_(*t*)

In this test, the 508 μm aluminum particle is used to generate sensor output signal *r*_1_(*t*) (Figure 11a) which also contains a vibration interference of 301 Hz from the vibration exciter shown in Figure 10.

**Figure 12.**
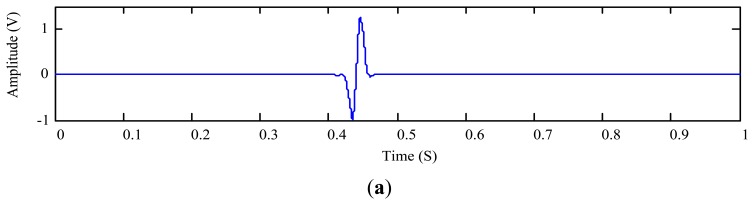

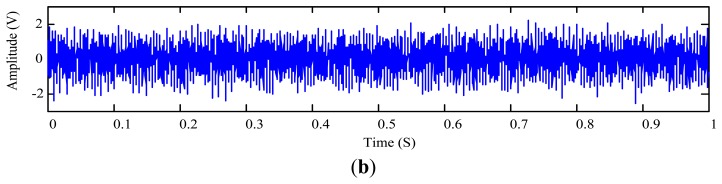
Extraction of the 508 μm aluminum particle signature using the proposed approach: (**a**) extracted particle signature; and (**b**) residue signal.

To test the proposed method under a lower SNR condition, another interference signal *r*_2_(*t*) is mixed into the collected signal. Hence the raw signal *x*(*t*) to be processed is given by:

(30) $$x(t)=r_{1}(t)+r_{2}(t)$$

where *r*_2_(*t*) = 0.5sin(2π400*t*)(1 + cos(2π40R(*t*)*t*) + 0.35sin(2π500*t*)(1 + cos(2π5R(*t*)*t*), *R*(*t*) is a random number varying between [0, 1]. The temporal waveform of *x*(*t*) is plotted in Figure 11b. The proposed MODWT-ODD approach is applied to measure the metallic particle signature which is shown in Figure 12a. The optimal decomposition depth is calculated as *L*_opt_ = 5 by employing the segment length of 80 data points (0.01 s); and the SNR is calculated using Figures 11b and 12a as −15.20 dB. After extracting the wear particle signature, one can plot the residue signal as shown in Figure 12b.

**Figure 13.**
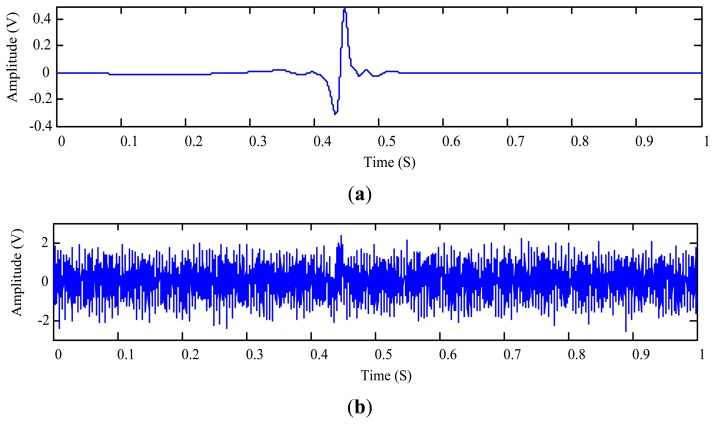
Extraction of the 508 μm aluminum particle signature using the reference approach [17]: (**a**) extracted particle signature; and (**b**) residue signal.

For comparison, we also employ the joint time-invariance wavelet transform and kurtosis approach [17] to process the same signal *s*(*t*). The decomposition depth used in the reference approach is calculated using Equation (16) as *L* = 10. With the wavelet-based approach from the reference, the extracted particle signature and the residue signal are obtained and displayed in Figure 13a,b, respectively.

Comparing Figure 12a with Figure 13a indicates that both the proposed and the reference approaches are capable of detecting the existence of the wear particle. The signature amplitude obtained using the reference approach with the maximum decomposition depth *L* is smaller. Nevertheless, one cannot confirm which measurement is more authentic because the ratio between the particle signature amplitude and the particle size is unknown. Fortunately, the residue signal can reflect the integrity of the extracted signature. As shown in Figure 12b, no footprint of the signature can be traced in the residue signal of the proposed approach. On the contrary, some remnants of the particle signature can be still identified from the residue signal yielded by the reference approach as shown in Figure 13b. To quantitatively evaluate the residue signals, it is calculated that the RMSDK_SSR of the proposed method is 0.032755, which is smaller than 0.033742 of the joint time-invariance wavelet transform and kurtosis approach. This suggests that the measured particle signature using the proposed approach is more authentic than the one obtained using the reference wavelet-based approach.

### 4.2. Metallic Particle Signature Extraction under Normal SNR Condition

**Figure 14.**
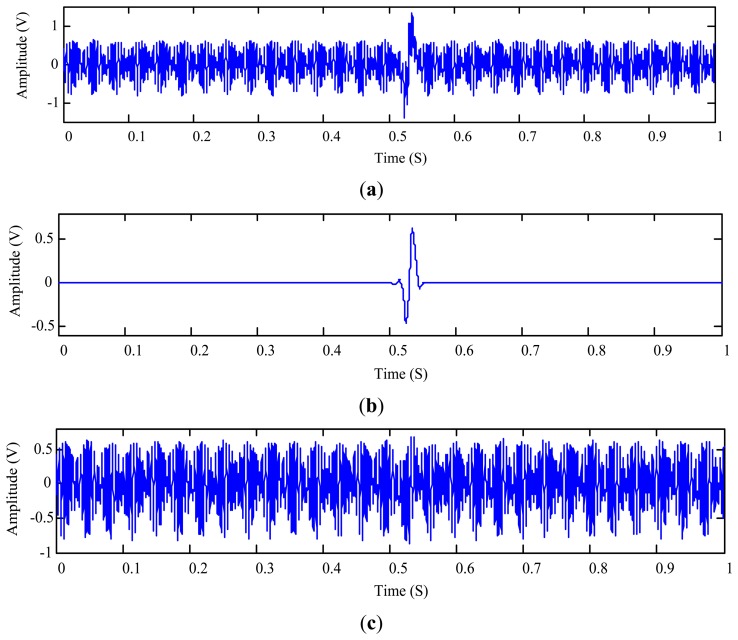
Extraction of the 125 μm aluminum particle signature: (**a**) sensor output signal; (**b**) extracted particle signature using the proposed approach; and (**c**) the residual signal.

In this case, the vibration interference frequency is arbitrarily set at 200 Hz and a 125 μm aluminum wear particle is used. The sensor output signal is shown in Figure 14a. The proposed approach is again used to find the optimal depth which is now *L*_opt_ = 6. The measured aluminum particle signature and the residue signal are displayed in Figures 14b,c, respectively. The associated SNR is −13.95 dB that is slightly higher than that in the previous test (Figure 11).

The RMSDK_SSR value of the residue signal shown in Figure 14c is 0.030438 which is lower than 0.031020 obtained by the joint time-invariance wavelet transform and kurtosis approach. This result again indicates that the proposed approach outperforms the reference approach in terms of the quality of the extracted particle signature.

### 4.3. Metallic Particle Signature Extraction under Higher SNR Condition

**Figure 15.**
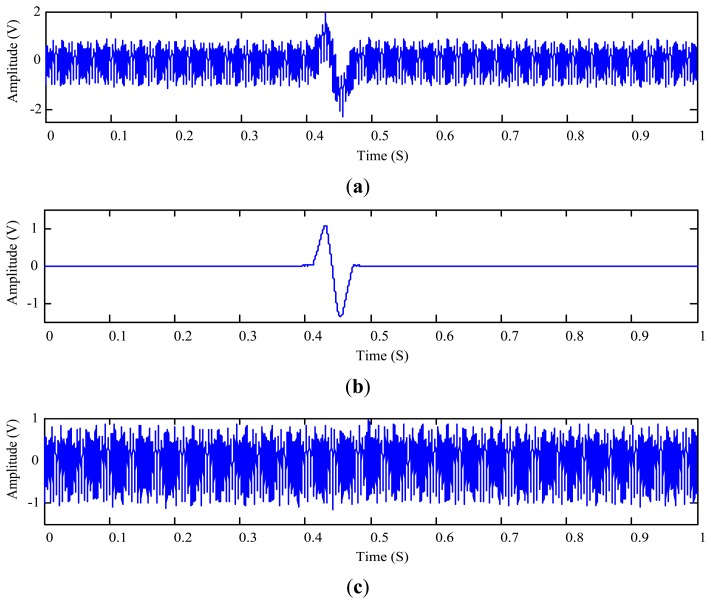
Extraction of the 70 μm titanium particle signature: (**a**) sensor output signal; (**b**) extracted particle signature; and (**c**) residue signal.

This test was done using a titanium wear particle of 70 μm in diameter. The interference created by the signal generator is randomly set at 905 Hz. The collected sensor output signal, the extracted particle signature and the residue signal obtained using the proposed approach are plotted in Figures 15a–c, respectively. The SNR is calculated as −8.43 dB from Figure 15.

Using the proposed approach, the optimal decomposition depth *L*_opt_ is found to be six levels, leading to a RMSDK_SSR value of 0.012122 for the residue signal as shown in Figure 15c. In comparison, the RMSDK_SSR value of the joint time-invariance wavelet transform and kurtosis approach is 0.012483. Again, the proposed approach is more effective in keeping the integrity of the measured particle signature.

Through comparing three experiments under different SNRs, one can see that both the proposed and the reference wavelet-based approaches are able to detect the wear particle existences for the oil debris sensors. However, the proposed method is more effective in maintaining the integrity of the measured signature due to the introduction of the optimal decomposition depth *L*_opt_.

As the particle signature is the representation of the wear particle in the oil debris system, the particle size can be reliably estimated based on the amplitude of the particle signature for the wear assessment. Our main concern in this paper is the software approach to enhance the wear particle monitoring capability of the oil debris sensor. The transduction between the particle signature and the particle size that is determined by the sensor structure is beyond our discussion. The details in this regard can be found in [36].

## 5. Conclusions

In this paper, a MODWT with optimal decomposition depth (MODWT-ODD) approach has been proposed to measure the wear particle signatures for the oil debris sensors. Considering the difficulty in directly measuring the level of integrity of the extracted signature in real applications, a RMSDK_SSR criterion was devised to evaluate the authenticity of the signature extraction indirectly via observing the short-term stationarity of the residue signal. In doing so, the RMSDK_SSR criterion was used to determine the optimal decomposition depth of the MODWT. The feasibility of using the RMSDK_SSR criterion to identify the optimal decomposition depth has been validated by the comparison with the Euclidean distance values in processing the simulated signal. The performance of the proposed method has also been examined using the real wear particle measurements. It is noting that the electromagnetic principle of the oil debris sensor is invalid to the non-metallic particles. Hence the proposed approach is only effective in wear monitoring of metallic moving parts. Our experiments indicate that the application of the MODWT at the optimal decomposition depth can monitor wear particle in lubricating oil more truthfully than the reference method.

## Figures and Tables

**Table 1.** Comparison between the Euclidean distance and the RMSDK_SSR.

**Decomposition Depth *j***	**Euclidean Distance *d*(*s*(*t*),*s****_j_***(*t*))**	**RMSDK_SSR *RMSDK_SSR*(*x*(*t*),*s****_j_***(*t*))**
2	5.65690	0.025946
3	5.65690	0.025946
4	0.82858	0.025590
5 (*L*_opt_)	0.65441	0.025581
6	0.85724	0.025607
7	1.48020	0.025709
8	1.26040	0.025657
9	1.26040	0.025657
10 (*L*)	1.31510	0.025658
